# Limited Flexion of the Middle Finger Due to a Degenerative Partial Flexor Tendon Rupture Mimicking a Soft Tissue Tumor

**DOI:** 10.7759/cureus.64534

**Published:** 2024-07-14

**Authors:** Koichi Yano, Tomoya Kato, Takuya Yokoi, Yasunori Kaneshiro, Teruo Kita

**Affiliations:** 1 Department of Orthopaedic Surgery, Seikeikai Hospital, Sakai City, JPN

**Keywords:** soft tissue mass, limited flexion, mechanical block, subcutaneous ruptures, flexor tendon rupture

## Abstract

Atraumatic subcutaneous rupture of the finger flexor tendon of the hand and forearm is rare. Most sites of closed and subcutaneous ruptures of the finger flexor tendon are the tendon-bone insertion and musculotendinous junction, and an intratendinous lesion is unusual. We report the case of a 76-year-old female who presented to our department with a one-month history of a soft tissue mass and limited flexion of the left middle finger without trauma. Preoperative magnetic resonance imaging revealed a soft tissue mass that caused limited finger flexion. Intraoperative findings showed an intratendinous rupture of the flexor digitorum profundus tendon at the middle phalanx; the lesion was resected to obtain smooth grinding of the tendon. One year postoperatively, the soft tissue mass and limited flexion of the finger resolved without recurrence.

## Introduction

Most closed and subcutaneous ruptures of the finger flexor tendons occur at the weakest sites of the tendons, including the tendon-bone insertion point and musculotendinous junctions [1]. Boyes et al. reported a series of 80 flexor tendon ruptures, with intratendinous rupture found in only three cases [2]. Atraumatic flexor tendon rupture is rare [3,4].

Limited finger flexion is caused by various pathologies, including stenosing tenosynovitis, infection, osteoarthritis, fracture, flexor tendon rupture, tumors, and neurological disorders.

Herein, we report a case of limited finger flexion due to a degenerative subcutaneous intratendinous flexor tendon rupture mimicking a soft tissue tumor and present preoperative and intraoperative photographs.

## Case presentation

A 76-year-old, right-handed female noticed a soft tissue mass without pain and limited flexion of the left middle finger without pain one month prior to presentation at our department. The patient visited our department because the symptoms did not improve. The patient’s occupation was janitorial services. The patient had no remarkable medical history, including steroid use and administration of intratendinous injection; in addition, the patient was not receiving antibiotics such as fluoroquinolones at the time of presentation. Furthermore, the patient could not remember any previous injury or popping or cramping sensations on the finger.

Initial physical examination revealed a soft tissue mass, 1 cm in diameter, on the volar side of the proximal interphalangeal (PIP) joint. There were no changes in skin color, no scarring, no tenderness, and no Tinel’s sign. The tumor was hard and did not adhere to the skin. In addition, the grip strengths of the right and left hand were 20.0 and 15.3 kg, respectively. The active extension/flexion ranges of motion of the left middle finger were as follows: metacarpophalangeal joint, 5°/70°; PIP joint, 5°/55°; and distal interphalangeal joint, −5°/40°. The passive range of the PIP joint was normal. The Disabilities of the Arm, Shoulder, and Hand (DASH) score was 16.4 for disability/symptom.

**Figure 1:**
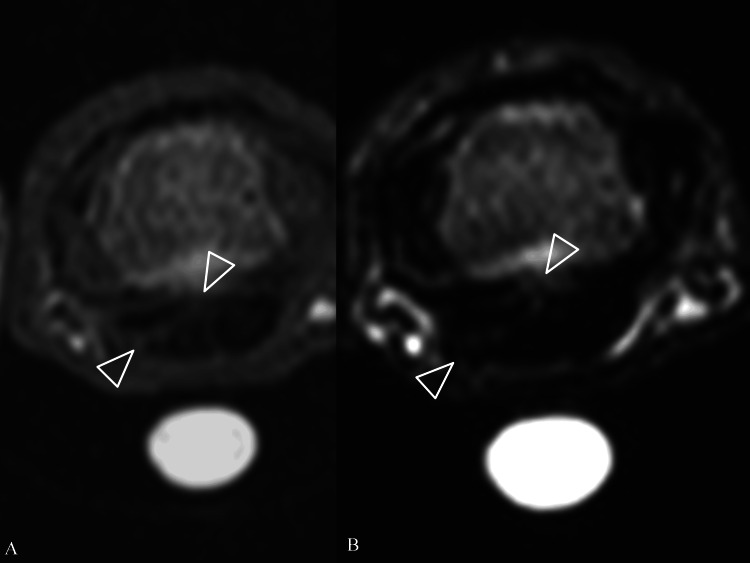
Preoperative magnetic resonance imaging (A) T1-weighted image. (B) T2-weighted image. The two white arrowheads indicate high signal intensity between the mass lesion and the flexor tendon.

Plain radiographs revealed no remarkable findings such as osteoarthritis or calcification. Ultrasound examination using power Doppler showed a low-echoic lesion next to the flexor tendon with no signal. Magnetic resonance imaging showed high signal intensity on T1- and T2-weighted images between the mass lesion on the ulnar side and flexor tendon, and the mass lesion showed low signal intensity on T1- and T2-weighted images (Figures 1A, 1B).

A radiologist interpreted the findings as a giant cell tumor in the tendon sheath. We added the fibroma and chronic hematoma as differential diagnoses. The preoperative diagnosis was a soft tissue tumor causing limited finger flexion due to a mechanical block.

**Figure 2:**
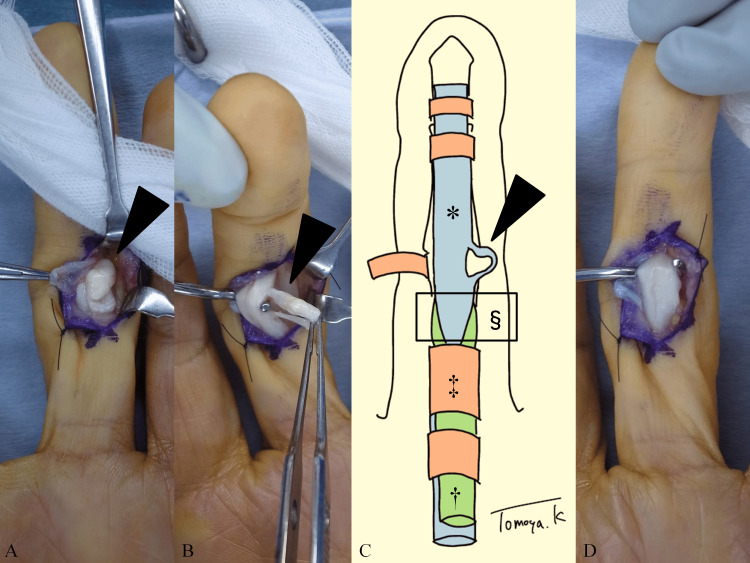
Intraoperative photographs and schema (A) Bulging lesions (black arrowhead) after opening the pulley. (B) Lesions (black arrowhead) connected to the proximal and distal sites of the flexor digitorum profundus (FDP) tendon. (C) Schema of (B). Pathological lesion (black arrowhead). FDP tendon (asterisk). Flexor digitorum superficialis tendon (dagger). A2-pulley (double dagger). Hiatus tendinous (section mark). (D) After lesion resection.

Surgery was performed under regional anesthesia using an air tourniquet. A zigzag incision was made, and the pulley was exposed. There was a bulging lesion under the A3-pulley, which was incised and opened. A white, round mass was observed (Figure 2A). When the tumor was pulled, it was found to be connected to the ulnar side of the flexor digitorum profundus (FDP) at the proximal and distal sites, similar to a ring (Figures 2B, 2C). On gross inspection, there was no crystal. This lesion was believed to cause limited motion due to entrapment; therefore, it was excised to achieve smooth tendon gliding (Figure 2D).

**Figure 3:**
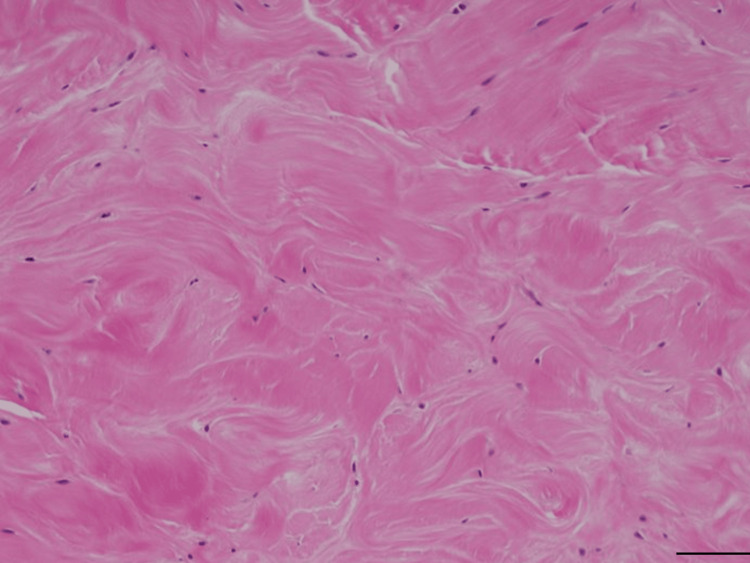
Histopathological analysis High-magnification hematoxylin and eosin staining of surgical specimens. Scale bar 100 mm.

After skin closure, a bulky dressing was applied. Active finger motion exercises were initiated the day after surgery. Histopathological examination of the lesion revealed a degenerative tendon structure, including loss of fiber arrangement, hyalinization, and rounding of the nuclei (Figure 3).

The patient was diagnosed with a degenerative intratendinous rupture of the FDP tendon.

At the final follow-up one year postoperatively, the patient was asymptomatic without recurrence and had no limitations in daily life and work. Grip strengths of the right and left hand were 22.2 and 18.7 kg, respectively. The active extension/flexion ranges of motion of the left middle finger were as follows: metacarpophalangeal joint, 15°/85°; PIP joint, 0°/110°; and distal interphalangeal joint, 0°/75°. The DASH score was 1.7 for disability/symptom and 0 for work.

## Discussion

We presented a case of limited flexion of the PIP joint of the finger due to a bulging lesion connected to the FDP tendon, which caused a mechanical block to the flexor digitorum sublimis (FDS) tendon at the hiatus tendinous or A2-pulley. The patient was asymptomatic after the lesion was resected. We diagnosed this lesion as a partial intratendinous flexor tendon rupture because histopathological examination revealed a degenerative tendon structure.

The flexor tendons of the finger are strong collagen cords. They connect the muscles of the forearm to the phalangeal bones in the fingers and thumb. There are two flexor tendons to each finger (FDS and FDP) and one to the thumb. The tendons run inside of the pulley system on the palm and finger, and they can flex fingers effectively. The most common cause of the flexor tendon rupture is trauma, and the tendons can be damaged by a cut. After flexor tendon rupture, the symptoms include the inability to flex the finger partially or fully, an extended finger position compared with the adjacent fingers at rest, and pain on flexion of the finger.

Similar lesions have been reported in patients with trigger fingers after trauma. Lee et al. reported six cases of trigger fingers caused by flexor tendon rupture after a puncture or laceration [5]. The interval between injury and surgery varied from 2 weeks to 12 months. All cases showed triggering and pain, unlike our case, and five of the six cases showed localized tenderness. Moreover, intraoperative photographs of the tendon showed a flap tear or T-shaped laceration, which differed from that in our case [5-7]. Our patient had no traumatic history or scar on the middle finger. Because the bulging lesion was connected to the FDP tendon at the proximal and distal ends like a ring, we assumed that this was caused by an intratendinous rupture along the direction of the tendon fiber.

Moreover, previous studies on the histological examination of spontaneous tendon ruptures of a tendon, including the Achilles tendon and biceps brachii, compared with a control specimen, showed degenerative changes in ruptured tendons [8]. Because pathological lesions were near the hiatus tendinous, a degenerative incomplete rupture might be caused by frequent hand use and attrition between the FDS and FDP. Lundborg et al. reported two avascular areas within the FDP tendon in Zone 2 due to watershed regions from the vascular supply [9]. Because the lesion in our case was near this area, intrinsic healing might be poor, and bulging lesions could occur.

Regarding treatment, Erhard et al. reported a biomechanical study of treatment for partial lacerations using cadaver FDP tendons [10]. In a 75% partial laceration model, treatment by trimming a partially lacerated tendon showed less gliding resistance and stronger tensile properties than treatment with a running suture. Therefore, we trimmed the bulging lesion to achieve smooth tendon gliding rather than a repair.

## Conclusions

Our case showed a one-month history of a soft tissue mass and limited flexion of the left middle finger without trauma. Preoperative images mimicked a soft tissue tumor at the middle phalanx. Intraoperative findings showed that the mass lesion was a subcutaneous flexor tendon rupture, and the lesion was resected to obtain smooth grinding of the tendon. One year postoperatively, the symptom resolved without recurrence. When a patient shows a soft tissue mass and limited flexion of the finger and preoperative images show a mass lesion within the pulley system, physicians must consider a non-traumatic subcutaneous rupture of the flexor tendon as a differential diagnosis.
